# Astragaloside IV as a Memory-Enhancing Agent: In Silico Studies with In Vivo Analysis and Post Mortem ADME-Tox Profiling in Mice

**DOI:** 10.3390/ijms25074021

**Published:** 2024-04-04

**Authors:** Katarzyna Stępnik, Wirginia Kukula-Koch, Anna Boguszewska-Czubara, Kinga Gawel

**Affiliations:** 1 Department of Physical Chemistry, Institute of Chemical Sciences, Faculty of Chemistry, Maria Curie–Skłodowska University in Lublin, Pl. M. Curie-Skłodowskiej 3, 20-031 Lublin, Poland; 2 Department of Pharmacognosy with Medicinal Plants Garden, Medical University of Lublin, 1 Chodzki St., 20-093 Lublin, Poland; virginia.kukula@gmail.com; 3 Department of Medical Chemistry, Medical University of Lublin, 4A Chodźki St., 20-093 Lublin, Poland; anna.boguszewska-czubara@umlub.pl; 4 Department of Experimental and Clinical Pharmacology, Medical University of Lublin, 8B Jaczewskiego St., 20-090 Lublin, Poland; kingagawel@umlub.pl

**Keywords:** astragaloside IV, passive avoidance, memory impairment, ADME-Tox, blood–brain barrier

## Abstract

Many people around the world suffer from neurodegenerative diseases associated with cognitive impairment. As life expectancy increases, this number is steadily rising. Therefore, it is extremely important to search for new treatment strategies and to discover new substances with potential neuroprotective and/or cognition-enhancing effects. This study focuses on investigating the potential of astragaloside IV (AIV), a triterpenoid saponin with proven acetylcholinesterase (AChE)-inhibiting activity naturally occurring in the root of *Astragalus mongholicus*, to attenuate memory impairment. Scopolamine (SCOP), an antagonist of muscarinic cholinergic receptors, and lipopolysaccharide (LPS), a trigger of neuroinflammation, were used to impair memory processes in the passive avoidance (PA) test in mice. This memory impairment in SCOP-treated mice was attenuated by prior intraperitoneal (ip) administration of AIV at a dose of 25 mg/kg. The attenuation of memory impairment by LPS was not observed. It can therefore be assumed that AIV does not reverse memory impairment by anti-inflammatory mechanisms, although this needs to be further verified. All doses of AIV tested did not affect baseline locomotor activity in mice. In the post mortem analysis by mass spectrometry of the body tissue of the mice, the highest content of AIV was found in the kidneys, then in the spleen and liver, and the lowest in the brain.

## 1. Introduction

Damage to the neurons in the human brain can cause several neurodegenerative diseases. The World Health Organization counts about 10 million new cases of dementia in the world every year [1]. It is widely recognized that aging is a key risk factor for dementia, as it leads to an accumulation of various unrepaired cellular damages as well as impaired cellular repair and compensation mechanisms [2,3]. It is estimated that by 2050, 16% of the world’s population will be seniors aged 65 or older. This is an increase of 7 percentage points compared to 2019. The trend of a rapidly aging society worldwide, the so-called silver tsunami, is important for the increase in cases of neurodegenerative diseases associated with cognitive impairment [4]. Neuroprotection is closely related to the rescue, regeneration and maintenance of the integrity of both neurons and the neurovascular unit to fulfill their physiological functions [5].

One of these diseases is Alzheimer’s disease (AD), a multifactorial and heterogeneous disorder characterized by a progressive loss of cognitive function [6]. The accumulation of protein aggregates in the brain in the form of insoluble amyloid plaques and neurofibrillary tangles (NFTs) is the pathological hallmark of AD [7]. Fibrillar amyloid β (Aβ) peptides, which are components of senile plaques, are formed by cleavage of the Aβ precursor protein, while the components of NFTs are the paired helical filaments of the abnormally phosphorylated microtubule-associated tau protein [6,8]. Currently, the release of amyloid β is thought to be the initiating event in the pathogenesis of Alzheimer’s disease, while NFTs are considered a secondary event in neurodegeneration [9]. Alzheimer’s disease can be caused by mechanisms of neuroinflammation [10] as well as neurotoxicity [11] or the induction of oxidative damage [12].

The cholinergic system is involved in many crucial physiological processes, e.g., learning and memory, attention, sleep, stress response and sensory information [13]. Cholinergic neurons, which innervate almost all areas of the brain [14,15] and therefore play a very important role in the peripheral and central nervous system, are particularly present in the spinal cord, hindbrain, medial habenula, mesopontine region, basal forebrain, striatum, olfactory tubercle and islets of Cajella complex [13,16,17].

In Alzheimer’s disease, the cholinergic neurons in the central nervous system (CNS) are degenerated, leading to a loss of function [17,18,19]. Acetylcholine (ACh), a neurotransmitter secreted by all cholinergic neurons, is associated with the memory process [20,21,22,23]. On the one hand, the attention deficit may be caused by injury to cholinergic neurons in the basal forebrain [24,25]; on the other hand, attention in humans can be improved by cholinesterase inhibitors that facilitate cholinergic neurotransmission [26,27]. In the treatment of senile dementia of the Alzheimer’s type, cholinergic receptor agonists (muscarinic and nicotinic) and inhibitors of the acetylcholine metabolizing enzyme or synthesis promoters are used to increase endogenous ACh levels [28]. However, the most clinically successful approach is the use of acetylcholinesterase inhibitors [28]. The search for new acetylcholinesterase inhibitors, including those from the plant kingdom, which could represent possible alternatives to the therapeutics currently in use, is an important issue in the treatment of AD. One of the plants that has the potential to improve cognitive functions, including memory and learning, is *Astragalus mongholicus.* In our previous paper, IC50 values were determined using the TLC bioautography assay [29], and blood–brain barrier (BBB) pharmacokinetic parameters for astragalosides I-IV (AI-AIV) were calculated using a computational analysis [30]. In addition, the logBB value (0.49 ± 0.03) of AIV was measured.

In our study, both the in vivo and post mortem studies were preceded by an analysis of ADME-Tox parameters characterizing the transport of a substance in a living organism, i.e., the L—liberation, A—absorption, D—distribution, M—metabolism, E—excretion and Tox—toxicity of a drug.

In this phase of the experiment, the passive absorption in the human jejunum, the Caco-2 absorption, the behavior towards P-glycoprotein and the toxicity risk were determined in silico. The ADME-Tox in silico profiling of AIV with regard to the transport of the active substance in the body was presented for the first time.

Both the Caco-2 and jejunum models were used to characterize passive absorption by the human intestinal epithelium. The Caco-2 cell monolayer, the human colonic epithelial cancer cell line, is an excellent model for the passive transcellular pathway [31]. The tight junctions between cells that form during Caco-2 cell differentiation serve as a model for the paracellular movement of substances through the intestinal monolayer [32]. Therefore, it can mimic the human intestinal epithelium and be compared to the extensively folded human jejunum [31]. To evaluate the accumulation of AIV in the selected internal organs, an LC-MS analysis of the kidney, spleen, liver and brain of mice was also performed after intraperitoneal administration of the substance.

In this study, the methodological pathway from the in silico ADME-Tox analysis of AIV to the post mortem studies in the soft tissues of mice was presented for the first time. The effect of attenuation of SCOP-induced memory impairment was thus confirmed, while toxic effects on the locomotor activity of mice could be excluded in in vivo tests.

## 2. Results

### 2.1. The ADME-Tox Studies

In our previous paper [30], promising results regarding the potential effects of astragalosides were presented. Therefore, we decided to continue this research and extend it to include in vivo testing in mice and the assessment of astragaloside IV accumulation in individual organs and tissues using both computational and post mortem analyses.

The ADME-Tox parameters calculated in silico are listed in Table 1 and Table S1 in the Supplementary Material section.

All tested astragalosides have poor or moderate passive absorption in the intestine. The lowest passive jejunum and Caco-2 permeability [cm/s] is shown by the two astragalosides III and IV (10^−7^ cm/s). This could be due to a larger number of H-donor groups compared to astragalosides I and II and/or the relatively low lipophilicity of the analytes.

### 2.2. The Influence of Astragaloside IV on the Acquisition and Consolidation of Long-Term Memory Impairment Induced by SCOP Administration in Mice

**Figure 1:**
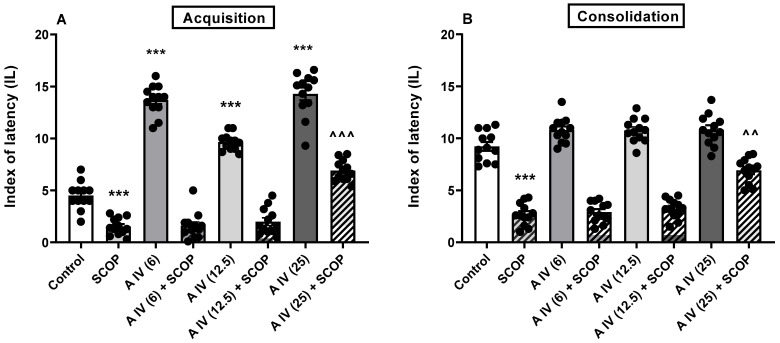
Effect of astragaloside IV (6, 12.5 or 25 mg/kg, ip) on acquisition (**A**) and consolidation (**B**) of long-term memory impairments induced by administration of SCOP (1 mg/kg, ip) in mice. Data are presented as individual measurements, mean ± SEM (n = 12 per group). *** *p* < 0.001 compared to control, ^^^ *p* < 0.001, ^^ *p* < 0.01 compared to SCOP. SCOP—scopolamine.

A one-way ANOVA revealed the differences between the tested groups in the animals for both the acquisition [F(7,88) = 192.5, *p* < 0.001; Figure 1A] and consolidation [F(7,88) = 116.5, *p* < 0.001; Figure 1B] of long-term memory impaired by SCOP administration. SCOP impaired both memory acquisition and consolidation compared to control mice (*p* < 0.001, Figure 1A,B). This memory impairment in SCOP-treated mice was attenuated by the earlier administration of astragaloside IV, but only at the highest dose, i.e., 25 mg/kg, ip (acquisition: *p* < 0.001 and consolidation: *p* < 0.01; Figure 1A,B). The smaller doses were ineffective (*p* > 0.05). Interestingly, astragaloside IV itself improved acquisition at all doses tested (*p* < 0.001; Figure 1A) but not the consolidation of long-term memory (*p* > 0.05; Figure 1B).

### 2.3. Influence of Astragaloside IV on the Acquisition and Consolidation of Long-Term Memory Impairments Induced by LPS Administration in Mice

**Figure 2:**
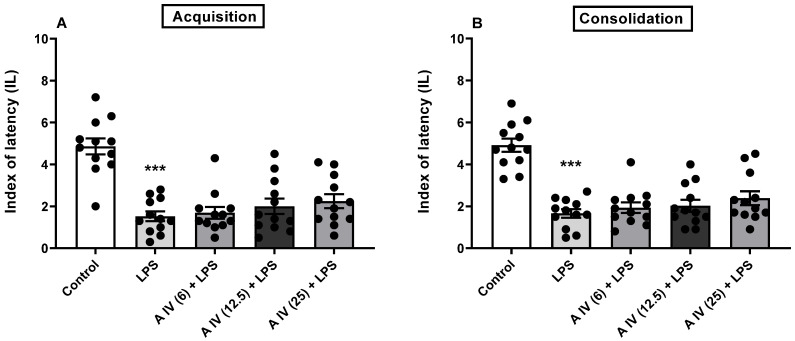
Effect of astragaloside IV (6, 12.5 or 25 mg/kg, ip) on the acquisition (**A**) and consolidation (**B**) of long-term memory impairment induced by LPS (2.5 mg/kg, ip) administration in mice. Data are presented as individual measurements, mean ± SEM (n = 12 per group). *** *p* < 0.001 compared to control. LPS—lipopolysaccharide.

The one-way ANOVA showed the differences between the tested groups in the animals for both the acquisition [F(4,55) = 17.65, *p* < 0.001; Figure 2A] and consolidation [F(4,55) = 22.49, *p* < 0.001; Figure 2B] of long-term memory impaired by LPS administration. LPS impaired both memory acquisition and consolidation compared to control mice (*p* < 0.001, Figure 2A,B). In all tested doses, astragaloside IV did not attenuate memory impaired by LPS in either the acquisition or consolidation phase (*p* > 0.05; Figure 2A,B) compared to mice treated with LPS alone.

### 2.4. Locomotor Activity

**Figure 3:**
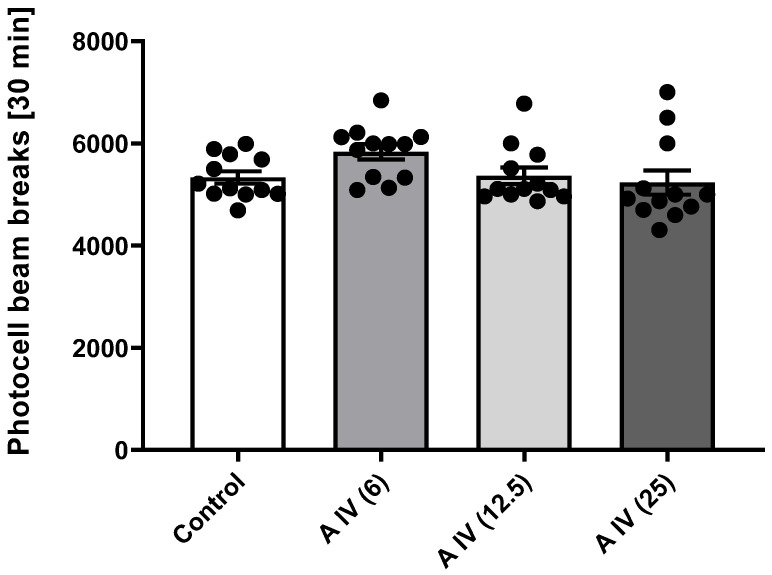
Effect of astragaloside IV (6, 12.5 or 25 mg/kg, ip) on locomotor activity. Data are presented as individual measurements, mean ± SEM. Locomotor activity was measured by interrupting the light beam of the photocell for 30 min (n = 12 per group).

The one-way ANOVA showed that astragaloside IV had no effect on the locomotor activity of the mice at the 30 min measurement in all doses tested [F(3,44) = 7.57, *p* > 0.05] (Figure 3).

### 2.5. Post Mortem Analysis of the Distribution of Astragaloside IV in the Soft Organs 

**Figure 4:**
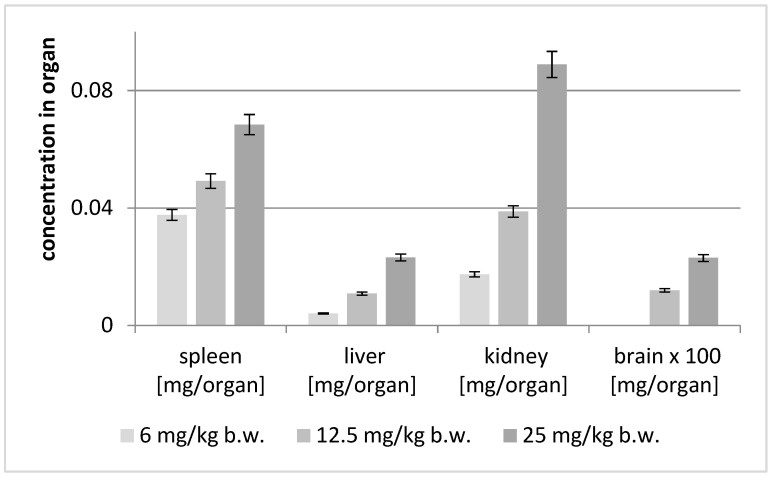
Concentration of astragaloside IV (mg/organ) after intraperitoneal injection of different concentrations of the compound.

**Figure 5:**
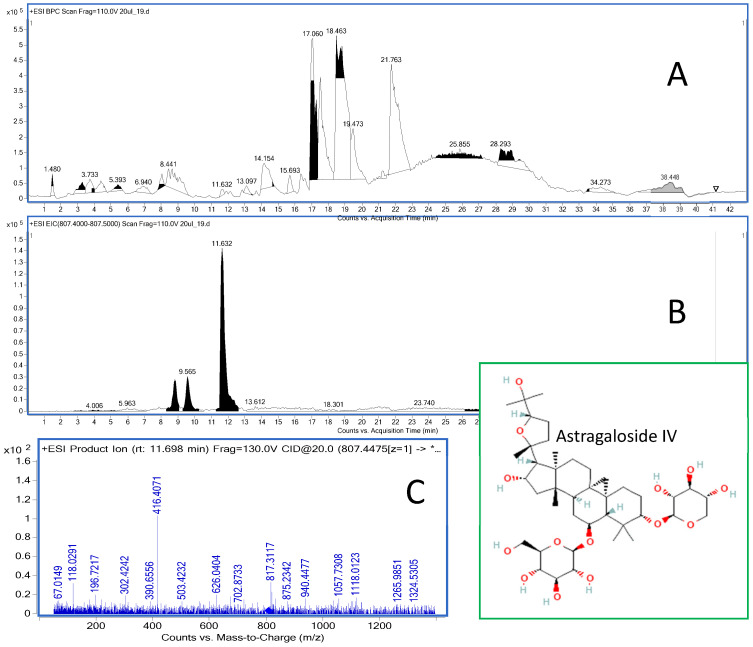
The total ion chromatogram of the liver extract recorded in positive ionization mode (**A**) together with the extracted ion chromatogram of astragaloside IV (11.6 min) (**B**) and the MS/MS chromatogram of the compound (**C**).

The analyses of the soft organs obtained from the experimental mice were performed using the HPLC-ESI-QTOF-MS/MS platform in the customized chromatographic method. The results of these studies are shown in Figure 4 and Table 2. In addition, the total ion chromatogram of the liver extract is shown in Figure 5.

The concentration of astragaloside IV in soft organ tissues was dose-dependent (see Figure 4). Interestingly, the highest content of the saponin was found in the kidneys, then in the spleen, liver and brain. At the dose of 25 mg/kg, the greatest concentration of the substance was observed in the kidneys, while at the lower doses the greatest concentration was found in the spleen.

**Figure 6:**
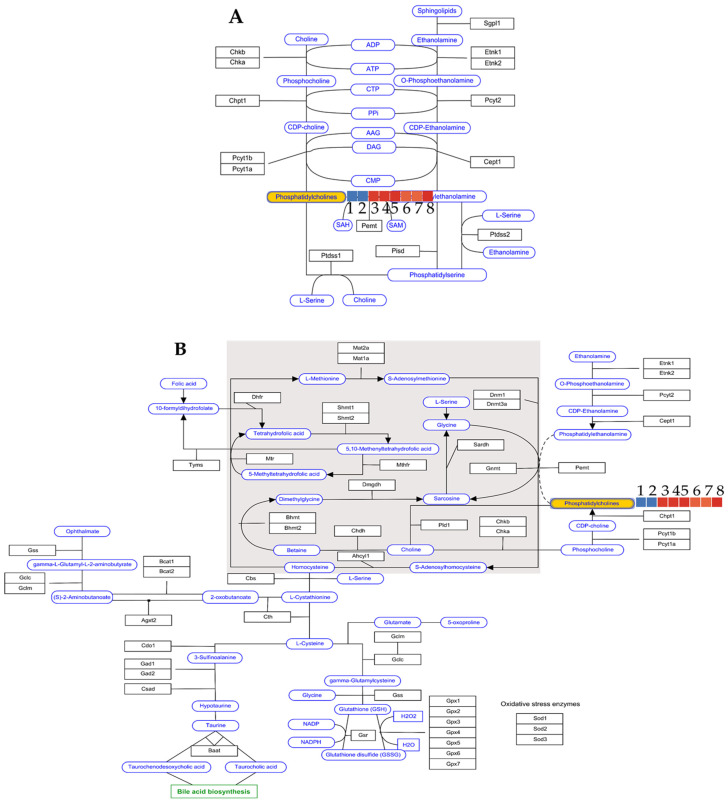
Effect of astragaloside IV treatment (25 mg/kg b.w. ip) on the levels of phosphatidylcholines as metabolic markers in the brains of mice involved in Kennedy metabolism (**A**) and one-carbon metabolism (**B**), expressed by log2 values compared to control groups (samples 1–8: see Table 3).

An analysis of the recorded mass chromatograms provided sufficient information to identify phosphatidylcholine in the extract. The compound eluted at 30.7 min was assigned the molecular formula C_46_H_85_NO_8_P and characterized—according to the METLIN database—as PC(22:4(7Z, 10Z, 13Z, 16Z)/16:0). The DB ID of the preliminarily identified metabolite was C00157 in the genome.jp database. When brain samples from three groups of animals—treated with saline, LPS and 25 mg/kg b.w. astragaloside IV—were analyzed, the log2 values differentiated the groups into two metabolic pathways: the Kennedy pathway and one-carbon pathway (see Figure 6A,B).

In both cases, the LPS model was found to decrease the level of phosphatidylcholines (PC) in the brains, whereas administration of astragaloside IV at a dose of 25 mg/kg b.w. ip restored the level of the compound to that of saline administration (see Table 3), thus reversing the decreasing effect of LPS on the metabolism of the synthesis of phosphatidylcholines.

## 3. Discussion

It should be emphasized that alternatives to animal testing, including computational (in silico) and biomimetic (non-cell-based in vitro) methods, should be used before any in vivo experimentation. For economic and ethical reasons, according to the European Union Directive 2010/63/EU of 22 September 2010, researchers are obliged to follow the 3Rs principle for laboratory animals and research methods, i.e., Replacement, Reduction, Refinement. Economic reasons relate primarily to the reduction in the use of chemical reagents and energy resulting from the commitment to the principles of green chemistry [33].

In silico computational methods have a screening character and are used upstream of research, mainly in combination with other methods, including biomimetic methods. Computational and biomimetic studies are performed before in vitro, in vivo and post mortem testing to determine specific parameters related to ADME processes [34]. The analysis of the values determined in silico from two different human intestinal absorption models (see Table 1) shows that there are no significant differences in the behavior of the compounds in relation to these two models. This means that the same compounds, namely astragalosides III and IV, have the lowest permeability in both the Caco-2 and jejunum models (4 × 10^−6^ and 6 × 10^−6^ cm/s, respectively), while astragalosides I and II have the highest absorbance. This could indicate that both absorption models are suitable for studying the intestinal absorption of the tested saponins in humans. This is consistent with the hypothesis that only the villus tips, which constitute part of the intestinal anatomical surface, are involved in the absorption process [35]. It is generally assumed that the incompletely and slowly passively absorbed substances are transported through the intestinal epithelium via the paracellular pathway through the water-filled pores. In drug research, it is assumed that the passage of the molecules through the cell barriers increases with increasing lipophilicity of the substance. However, some differences in intestinal absorption have been observed where the excessive lipophilicity of a compound can lead to absorption in the small intestine [36]. On the other hand, it is also possible that even very hydrophilic compounds can be transported through the intestinal barrier mainly by the transcellular route [37]. It is also assumed that the optimal transepithelial passage of drugs occurs when the n-octanol/water partition coefficient, which represents the lipophilicity descriptor, is about 3 [36]. In our case, the logPow values of astragalosides III and IV are in this optimal range, while the values of astragalosides I and II are much higher. It should also be borne in mind that the compounds remain longer in the intestinal lumen before they are absorbed. Therefore, they can diffuse further into the length of the intestinal villi compared to the compounds that are highly permeable through the intestinal endothelium and are rapidly and completely absorbed [38].

The P-glycoprotein, which belongs to the ATP-binding cassette transport proteins, plays an important role as an efflux pump by translocating various endogenous and exogenous substances into the extracellular compartment [39]. This protein with a mass of 170 kD is produced by the multidrug resistance 1 gene. In addition, it is a major component of the BBB that may be essential for brain detoxification and protection against xenobiotics [6]. The expression of P-gp can lead to a reduced permeability of the BBB [40], resulting in protection of the brain from toxic substances. However, this effect can also lead to reduced efficacy in the treatment of neurodegenerative diseases. The drugs that are P-gp substrates can drastically reduce the permeability of the BBB [6], so that the therapeutic effect cannot be achieved.

The in silico studies also estimated the probability that astragalosides are P-gp substrates. In our case, astragalosides I and II are likely to be P-gp substrates, while astragalosides III and IV are not. This could be due to the number of H-acceptors, which mainly determines the classification of the compound as a P-gp substrate [41]. However, as shown in Table 1, the differences between these values are not significant. In addition, other physicochemical descriptors that characterize a molecule determine whether it is a P-gp substrate or not. These parameters also include molecular size, expressed by molar weight or volume, and ionization, indicated by the pKa values for acid and base [41]. No significant differences were found for these parameters either. However, when the lipophilic logPow parameter and the polarizability of the molecule are taken into account, astragalosides I and II were found to be the most lipophilic compared to astragalosides III and IV and have higher values for the topological polar surface area. Therefore, the prediction of P-gp substrate specificity is a difficult task due to the various factors characterizing the molecules. However, considering the risk assessment of adverse effects performed for astragalosides using the OSIRIS Property Explorer recommended by the U.S. Food and Drug Administration (4/28/23), no mutagenic, tumorigenic, irritant or reproductive adverse effects were identified. This indicates that all compounds tested may be safe for human health.

Here, SCOP was used to impair memory processes in the PA test in mice. Scopolamine is an antagonist with a high affinity to cholinergic muscarinic receptors that can easily cross the BBB [42] and causes cognitive impairment in humans, rodents and non-human primates [43]. The blockade of muscarinic receptors by SCOP mimics the age-related degeneration of cholinergic neurons in the basal forebrain [44,45,46,47], which is why SCOP is often used as a model for cognitive impairment in vivo [48,49,50,51].

In our study, pretreatment with astragaloside IV at the highest dose reversed the SCOP-induced memory impairment in the PA task in mice. In our previous studies [29], astragaloside IV was shown to be an inhibitor of AChE, the enzyme responsible for the degradation of ACh in the synaptic cleft [52]. From this, it can be concluded that astragaloside IV reduces, at least in part, the impairment of memory by muscarinic receptors by increasing the affinity of ACh for muscarinic and probably also for nicotinic cholinergic receptors. It can be assumed to act in the same way as the drugs used to treat Alzheimer’s disease, namely donepezil, rivastigmine and galantamine—all acetylcholinesterase inhibitors [53,54].

In this study, pretreatment with astragaloside IV did not reverse the effect of the widely used neuroinflammation model [55]—LPS at any dose. Therefore, it can be concluded that it is very likely that it does not reverse memory impairment through anti-inflammatory mechanisms.

Astragaloside IV was detected in positive ionization mode in all biological samples as a signal with an *m*/*z* of 817.4570 Da. At this measured *m*/*z* value, the mass error was only 1.24 ppm. The compound was characterized by the double bond equivalent value of 8, which also supported its identity. As mentioned above, the effect of intraperitoneal administration of astragaloside IV at a dose of 25 mg/kg b.w. on phosphatidylcholine levels was observed. Administration of the drug reverses the negative effect of LPS on the metabolism of phosphatidylcholine, as shown by the log2 values of the Kennedy and one-carbon pathways (see Table 3). Marcucci et al. [56] demonstrated a proneurogenic effect of phosphatidylcholine, which contributes to increasing the population of healthy neurons. In addition, phosphatidylcholine attenuates inflammation-induced neuronal damage and consequently influences neuronal plasticity. The results of our study also confirm these observations.

Post mortem analysis of the mice tissue is part of the ADME-Tox profile. The high concentration of AIV in the spleen tissue of mice could indicate that this saponin can impair the function of the organ. The spleen, as a peripheral immune organ, is involved in the production of immune cells that act as immune effectors, including macrophages, monocytes, T cells, B cells and neutrophils [57]. Numerous scientific studies have shown that astragaloside IV can control immune cells such as macrophages, natural killer cells or lymphocytes as well as influence the production of various cytokines and chemokines [58]. In addition, AIV has been shown to be able to alleviate polymicrobial sepsis and pathological splenic damage, probably due to the inhibition of the inflammatory response and apoptosis of lymphocytes [59].

An analysis of the astragaloside IV concentration in the kidneys of mice suggests that excretion of astragaloside IV may occur in the urine. However, the study by Zhang et al. [60] shows that AIV can also be excreted slowly via hepatic clearance. The lowest amount of astragaloside IV was found in the brain. The method used could not detect the saponin in the mice treated with 6 mg/kg, as the measured value was too low to provide meaningful data. In our previous work [30], the concentration of AIV in the brain tissues was measured to calculate the logBB value, which is expressed as the logarithmic ratio between the concentration of a compound in the brain and in the blood. The calculation of this value is particularly important for the early estimation of BBB permeability in drug development. Taking into account the experimental value (0.49 ± 0.03), it can be concluded that astragaloside can cross the blood–brain barrier, but this passage can be impeded and slowed down. This may be due to the molecular weight of the compound of 785 g/mol and/or the topological polar surface area (TPSA) of 228.22 Å^2^ (data calculated with ACD/Percepta software, version 2012, Advanced Chemistry Development, Inc., Toronto, ON, Canada). In addition to lipophilicity, TPSA is extremely important for the ability of the drug to cross the BBB [61,62]. It is reported that the TPSA for orally bioavailable drugs should be less than 120 Å^2^ [63]. It is also known that the CNS drugs characterized by good brain penetration have a PSA of <100 Å^2^ or even smaller, <60–70 Å^2^ [63]. Nevertheless, the ability of astragaloside IV to cross the blood–brain barrier in the post mortem studies that we have demonstrated [30] is not inconsistent with the values of the physicochemical parameters mentioned above. There is also another theory that relates BBB permeability to the molecular flexibility [64,65] of a compound, which can be expressed, for example, by the number of rotatable bonds [66]. According to this theory, the more flexible molecules with a comparable molecular weight seem to adapt better to the membrane than the rigid ones [65]. Although the data obtained show some trends in the ADME profile of the saponin, they were collected after a single injection of the compound in question. Therefore, further studies should be conducted to obtain conclusive results.

## 4. Materials and Methods

### 4.1. Isolation of Astragaloside IV and ADME-Toxicity Profiling

The roots of *Astragalus mongholicus* were obtained from Ulaanbaatar (Bayangol district) in July 2017. They were authenticated by Dr. Otgonbataar Urjin from the Mongolian National University of Medical Sciences. The extraction procedure is described in our previous paper [29]. The saponins tested were identified according to the protocol described in the European Pharmacopea 8.0 Edition. The isolated astragaloside IV with a purity of 93.5% obtained according to the previously described protocol was subjected to the tests described here [30].

In brief, a 50% ethanolic extract was obtained from the roots of *Astragalus mongholicus* by ultrasound-assisted extraction. The plant material was weighed, poured with 50% ethanol and extracted in an Erlenmeyer flask in a solid to solute ratio of 1:5 using ultrasonic bath for 15 min. Three replicates were carried out with the same plant material, and the extracts were combined and evaporated to dryness in a rotary evaporator at 45 °C. The obtained dried residue was used for the isolation of AI-AIV by preparative HPLC chromatography. First, the extract was resuspended in 10% acetonitrile, filtered through nylon syringe filters (pore diameter 0.2 µm) and then subjected to HPLC fractionation in a gradient of acetonitrile in water—from 10% to 60% within 1 h. The purity of the fractions was checked by HPLC-ESI-QTOF-MS/MS chromatography and the AI-AIV-rich fractions were collected and evaporated to dryness.

The ADME-Tox studies were performed on the triterpenoid saponins from *Astragalus mongholicus* roots (AI-AIV) with the highest demonstrated neuroprotective potential [30]. To evaluate the ADME-Tox properties, some physicochemical parameters of AI-AIV were calculated in silico using ACD/Percepta software (version 2012, Advanced Chemistry Development, Inc., Toronto, ON, Canada). The following values were calculated: the logarithm of the n-octanol/water partition coefficient (logPow), the absorption in the human jejunum, the Caco-2 absorption, the number of H-donors and H-acceptors and the behavior towards P-glycoprotein. In addition, the toxicity risk, i.e., mutagenic, tumorigenic, irritant and reproductive potency, was estimated using the Osiris Property Explorer online.

### 4.2. Animal Studies

#### 4.2.1. Mice

Male adult Swiss mice (weight 25–35 g) were housed at the Centre for Experimental Medicine of the Medical University of Lublin, Poland. The mice were housed in groups of 5 animals per cage under standard environmental conditions (12:12 h light–dark, room temperature 21 ± 1 °C). The animals had ad libitum access to tap water and mouse food (Agropol, Motycz, Poland). They were acclimatized to the laboratory conditions for at least one week prior to the experiments. Each experimental group consisted of 12 animals. Throughout the experiments, every effort was made to minimize animal suffering and reduce the number of animals. The behavioral experiments were conducted regularly between 8:00 am and 3:00 pm, in accordance with the National Institute of Health guidelines for the care and use of laboratory animals and on the basis of the Council Directive of the European Community for the care and use of laboratory animals of 22 September 2010 (2010/63/EU). The experiments were approved by the Ethics Committee of the University of Life Sciences in Lublin, Poland (No. 45/2018). Each mouse was used only once for the experiment.

#### 4.2.2. Medication

Astragaloside IV (6, 12.5 or 25 mg/kg), isolated from the extract of *Astragalus mongholicus* roots [30], was placed in a mortar and dissolved in physiological saline (0.9% NaCl) with the addition of 0.2% dimethyl sulfoxide (DMSO). SCOP (Sigma Aldrich, St. Louis, MO, USA; 1 mg/kg) and LPS (Sigma Aldrich, St. Louis, MO, USA; 2.5 mg/kg) were dissolved in 0.9% NaCl (saline). The control groups received injections of saline with the addition of 0.2% DMSO. All preparations were administered intraperitoneally at a volume of 10 mL/kg b.w. Fresh drug solutions were prepared on each day of the study.

#### 4.2.3. The Passive Avoidance Test

The passive avoidance test in mice is used to assess fear-related learning and memory [67,68]. The apparatus consists of an acrylic box with two chambers: white (10 × 13 × 15 cm) and black (25 × 20 × 15 cm). During the experiment, the white chamber is illuminated with a bright light (8 W). The black chamber, which is equipped with an electric grid base, is connected to the illuminated chamber.

A pre-test (training) was carried out before the experiments. Each mouse was placed individually in the illuminated chamber of the device and allowed to explore it within 30 s. The guillotine door separating the two chambers was then opened. The mouse was then allowed to enter the black chamber, where it was immediately treated with an electric shock (0.2 mA) for 2 s. The latency to enter the black chamber was recorded (TL1). During the retention trial, which was performed 24 h after the pre-test, the same mouse was again placed in the lighted chamber of the device. The time required to enter the black chamber was recorded (TL2). In contrast to the pre-test, no foot shock was administered this time. If the mouse did not enter the black chamber within 300 s, TL2 was recorded as 300 s. The index of latency (IL) was calculated as follows:

IL = (TL2 − TL1)/TL1(1)

1. *Acquisition of long-term memory*

Astragaloside IV (6, 12.5 or 25 mg/kg, ip) was injected 15 min before administration of SCOP or LPS. After the 30 min delay, the pre-test was performed.

2. *Consolidation of long-term memory*

Astragaloside IV (6, 12.5 or 25 mg/kg, ip) was injected immediately after the pre-test, SCOP or LPS 30 min later.

#### 4.2.4. The Locomotor Activity Test

The locomotor activity of the mice was measured with the photoresistance actimeters (round cages, 25 cm diameter, two light beams), which were set up in the sound-attenuated test room. Before the test, the mice were acclimatized to the new environment for at least one week. The animals were placed individually in the actimeters for 30 min. Two photocells, mounted perpendicular to the axis, automatically measured the beam interruptions. During the experiment, the animals were divided into the following drug groups: the control group and the astragaloside IV group (6, 12.5 or 25 mg/kg, ip). Immediately after the injection, the animals were placed in the actimeters.

#### 4.2.5. Statistical Analysis

Behavioral data were analyzed using one-way analysis of variance (ANOVA) and Tukey’s post hoc test. GraphPad Prism 9.3.1 software (San Diego, CA, USA) was used for statistical purposes. A *p*-value < 0.05 was considered statistically significant. Data are presented as individual measurements, mean ± standard error of the mean (SEM).

### 4.3. HPLC-ESI-QTOF-MS/MS Determination of Astragaloside IV in the Tissues of the Animals

The mice were decapitated 0.5 h after completion of the locomotor activity test (i.e., 1 h after astragaloside IV injection) and the brains, livers, kidneys and spleens were collected, frozen and stored for quantitative analysis. They were then homogenized in an Eppendorff plastic vial kept in ice. The homogenates of the organs were vortexed with 200 µL of 50% ethanol for 10 min. The Eppendorff vials were centrifuged at 3500 rpm for 5 min and the supernatant was filtered through a nylon syringe filter with a diameter of 0.22 µm into the autosampler vials with the glass inserts. Astragaloside IV was dissolved in 50% ethanol and transferred to 10 different concentrations in the range of 0.002–0.2 mg/mL to obtain the calibration curve. Due to the small size and mass of brains, AIV was quantified in these organs after addition of the reference compound (Sigma Aldrich, St. Louis, MO, USA) to the samples. For this purpose, 100 µL of 0.5 mg/mL AIV was added to each brain sample. The peak areas of astragaloside IV in the brains were compared with the blank solution of the standard compound. The levels of astragaloside IV in the other organs were measured without the presence of the reference compound.

The detection of astragaloside IV in the brains, spleen, liver and kidneys of the mice was performed using a liquid chromatograph (1200 series) with mass detector (6500 series), the HPLC-ESI-QTOF-MS/MS instrument from Agilent Technologies (Santa Clara, CA, USA). The liquid chromatograph consisted of a degasser, a binary pump, a column oven, an autosampler and the high-resolution mass detector QTOF-MS/MS with an electrospray ionization source. The chromatographic column Zorbax Eclipse Plus (Agilent Technologies, CA, USA) (150 mm × 2.1 mm, 3.2 um, RP-18) was used to separate the metabolites of the injected matrix in the following gradient of acetonitrile with 0.1% formic acid (solvent B) in the 0.1% aqueous solution of formic acid (solvent A): 0 min—2% B, 2 min—10% B, 6 min—40% B, 35–37 min—95% B and 37.5 min—2% B. The analysis time was set to 43 min, the follow-up time to 5 min, the flow rate to 0.2 mL/min and the injection volume to 10 μL. The detailed settings of the mass spectrometer were as follows: a capillary voltage of 3000 V, a skimmer voltage of 65 V, a mass range of 100–1200 *m*/*z*, a collision energy of 10 and 20 V, a nebulizer pressure of 30 psig, a gas flow rate of 12 L/min and gas and sheath gas temperatures of 250 and 325 °C. The Mass Hunter Workstation (version B.10.00) from Agilent Technologies was used to record and process the data obtained. For quantitative analysis, the calibration curve equation of astragaloside IV (8 points) was generated from the triplicate injections ranging from 0.0001 to 0.1 mg/mL (n = 3). For each of the concentrations, the average values of the content of the tested substance in the respective organ were calculated. The standard deviation (SD) values were calculated in each case. The recorded mass chromatograms for the brain samples (injection with saline: n = 2, injection with LPS: n = 3 and 25 mg/kg b.w. with LPS injection: n = 3) were analyzed with the Mass Profiler Professional Program 15.1 from Agilent Technologies (Santa Clara, CA, USA) with a built-in pathways analysis module that uses the Kyoto Encyclopedia of Genes and Genomes (KEGG) database to detect the effects of the injected substance on molecular mechanisms. First, the raw data from the recorded chromatograms were exported in csv format and analyzed in the program. The PCA analysis carried out with the data set showed clear differences between the samples. Of the 517 entities analyzed, eleven differentiating *m*/*z* signals were detected in the samples, with *p* < 0.05 and a fold change of 2 settings. One of the differentiating signals was phosphatydylcholine, whose behavior in the tested samples was analyzed with the pathways module.

## 5. Conclusions

The memory impairment caused by intraperitoneal administration of SCOP was attenuated by the AIV dose of 25 mg/kg. The AIV doses of 6 and 12.5 mg/kg had no effect on the acquisition and consolidation of long-term memory impaired by SCOP in mice. None of the astragaloside IV doses attenuated the memory impairment induced by LPS administration. However, the effect of astragaloside IV on memory impairment induced by other inflammatory agents should be investigated to draw a definitive conclusion. The results of the in silico ADME-Tox studies on astragaloside IV indicate that its use is probably safe. The highest levels of the saponin were found in the kidneys, then in the spleen and liver, and the lowest in the brain. Astragaloside IV appears to be an efficient drug candidate for the treatment of CNS disorders with memory impairment.

## Figures and Tables

**Table 1:** The logarithm of the n-octanol/water partition coefficient (logPow), the logarithm of the brain/blood partition coefficient (logBB), the passive human jejunum absorption (Pe,jejunum), the Caco-2 absorption (Pe,Caco2), the number of H-donors, the number of H-acceptors, the P-glycoprotein substrate: (+) high probability and (−) low probability of being P-gp substrate, calculated in silico using ACD/Percepta software (version 2012, Advanced Chemistry Development, Inc., Toronto, ON, Canada).

Name	logPow	logBB	Pe,jejunum [10^−4^ cm/s]	Pe,Caco-2 [10^−6^ cm/s]	Number of H-donors	Number of H-acceptors	P-gp Substrate
AI	5.020	0.46	1.05	2.0	7	16	+
AII	4.459	0.11	0.25	0.5	8	15	+
AIII	3.767	0.15	0.04	0.1	9	14	-
AIV	3.757	0.49 *	0.06	0.1	9	14	-

* experimental value [30].

**Table 2:** Content of astragaloside IV in the tested organs of mice depending on the administered dose (ip). (Av—the average value, SD—the standard deviation, n = 3; the colors used represent the differences in content between the samples, with the green color representing the highest value obtained and the red color the lowest value).

	Brain [mg/Organ]		Spleen [mg/Organ]		Liver [mg/Organ]		Kidney [mg/Organ]	
Animal Group	Av	SD	Av	SD	Av	SD	Av	SD
6 mg/kg	ND	ND	0.03766	0.00186	0.00411	0.00011	0.01740	0.00209
12.5 mg/kg	0.00012	0.00001	0.04919	0.00093	0.01084	0.00023	0.03882	0.00092
25 mg/kg	0.00023	0.00002	0.06844	0.00133	0.02316	0.00016	0.08892	0.00613

**Table 3:** The results of the log2 values calculated for the phosphatidylcholine level (C_46_H_85_NO_8_P, PC(22:4(7Z, 10Z, 13Z, 16Z)/16:0) of brain samples analyzed without and with astragaloside IV administration.

Group Number	Group Type	Kennedy Pathway log2	One-Carbon Metabolism log2	Number of Brains Tested
1	LPS brain	0	0	2
2	LPS brain	0	0	2
3	Astragaloside IV 25 mg/kg	20.425	20.425	3
4	Astragaloside IV 25 mg/kg	20.611	20.611	3
5	Astragaloside IV 25 mg/kg	20.763	20.763	3
6	Saline group	18.991	18.991	3
7	Saline group	18.766	18.766	3
8	Saline group	20.984	20.984	3

## Data Availability

Data is contained within the article and Supplementary Material.

## Supplementary Materials

The supporting information can be downloaded at: https://www.mdpi.com/article/10.3390/ijms25074021/s1.

## Abbreviations

AIV—astragaloside IV; ACh—acetylcholine; AChE—acetylcholinesterase; AD—Alzheimer’s disease; ADME-Tox—absorption, distribution, metabolism, excretion and toxicity; Aβ—amyloid β; BBB—blood–brain barrier; CNS—central nervous system; DMSO—dimethyl sulfoxide; HPLC-MS—high-performance liquid chromatography coupled with mass spectrometry; IL—index of latency; ip—intraperitoneal administration; LPS—lipopolysaccharide; NFTs—neurofibrillary tangles; PA—passive avoidance; P-gp—P-glycoprotein; SCOP—scopolamine; SD—standard deviation; SEM—standard error of the mean; TPSA—topological polar surface area.
