# Supplementary material for: Astragaloside IV as a Memory-Enhancing Agent: In Silico Studies with In Vivo Analysis and Post Mortem ADME-Tox Profiling in Mice
Source: Int J Mol Sci. 2024 Apr 4;25(7):4021. doi: 10.3390/ijms25074021 (PMC11012721; doi:10.3390/ijms25074021)
Supplement: Supplementary file 1 [file ijms-25-04021-s001.zip › ijms-2937415-supplementary.pdf]

Table S1. Risk assessment of adverse side effects by OSIRIS Property Explorer of the tested astragalosides.

Name	mutagenic	tumorigenic	irritant	reproductive effective
A I	■	■	■	■
A II	■	■	■	■
A III	■	■	■	■
A IV	■	■	■	■

■ no risk
